# Epidemiology of foot-and-mouth disease in Landhi Dairy Colony, Pakistan, the world largest Buffalo colony

**DOI:** 10.1186/1743-422X-5-53

**Published:** 2008-04-29

**Authors:** Joern Klein, Manzoor Hussain, Munir Ahmad, Muhammad Afzal, Soren Alexandersen

**Affiliations:** 1 National Veterinary Institute, Technical University of Denmark, Lindholm, DK-4771 Kalvehave, Denmark; 2 Norwegian University of Science and Technology, Faculty of Medicine, Department of Cancer Research and Molecular Medicine, N-7489 Trondheim, Norway; 3 Food and Agriculture Organization of the United Nations – Pakistan, NARC, Park Road, PK-45500, Pakistan; 4 Ministry of Food, Agriculture & Livestock Pakistan, Livestock wing, PK-44000, Pakistan

## Abstract

**Background:**

Foot-and-mouth disease (FMD) is endemic in Pakistan and causes huge economic losses. This work focus on the Landhi Dairy Colony (LDC), located in the suburbs of Karachi. LDC is the largest Buffalo colony in the world, with more than 300,000 animals (around 95% buffaloes and 5% cattle, as well as an unknown number of sheep and goats).

Each month from April 2006 to April 2007 we collected mouth-swabs from apparently healthy buffaloes and cattle, applying a convenient sampling based on a two-stage random sampling scheme, in conjunction with participatory information from each selected farm. Furthermore, we also collected epithelium samples from animals with clinical disease, as well as mouth-swabs samples from those farms. In addition, we analysed a total of 180 serum samples randomly collecting 30 samples each month at the local slaughterhouse, from October 2006 to March 2007.

Samples have been screened for FMDV by real-time RT-PCR and the partial or full 1D coding region of selected isolates has been sequenced. Serum samples have been analysed by applying serotype-specific antibody ELISA and non-structural proteins (NSP) antibody ELISA.

**Results:**

FMDV infection prevalence at aggregate level shows an endemic occurrence of FMDV in the colony, with peaks in August 2006, December 2006 and February 2007 to March 2007. A significant association of prevalence peaks to the rainy seasons, which includes the coldest time of the year and the muslimic Eid-festival, has been demonstrated.

Participatory information indicated that 88% of all questioned farmers vaccinate their animals.

Analysis of the serum samples showed high levels of antibodies for serotypes O, A, Asia 1 and C. The median endpoint-titre for all tested serotypes, except serotype C, in VNT titration is at a serum dilution of equal or above 1/100.

All 180 serum samples collected have been tested for antibodies against the non-structural proteins and all but four have been found positive.

Out of the 106 swab-samples from apparently healthy and affected animals positive in real-time RT-PCR, we sequenced the partial or full 1D coding region from 58 samples. In addition we sequenced the full 1D coding region of 17 epithelium samples from animals with clinical signs of FMD. From all sequenced samples, swabs and epithelium, 19 belong to the regional PanAsia II lineage of serotype O and 56 to the A/Iran/2005 lineage of serotype A.

**Conclusion:**

For an effective and realisable FMD control program in LDC, we suggest to introduce a twice annually mass vaccination of all buffaloes and cattle in the colony. These mass vaccinations should optimally take place shortly before the beginning of the two rainy periods, e.g. in June and September. Those vaccinations should, in our opinion, be in addition to the already individually performed vaccinations of single animals, as the latter usually targets only newly introduced animals. This suggested combination of mass vaccination of all large ruminants with the already performed individually vaccination should provide a continuous high level of herd immunity in the entire colony.

Vaccines used for this purpose should contain the matching vaccine strains, i.e. as our results indicate antigens for A/Iran/2005 and the regional type of serotype O (PanAsia II), but also antigens of the, in this world region endemic, Asia 1 lineage should be included.

In the long term it will be important to control the vaccine use, so that subclinical FMD will be avoided.

## Background

Foot-and-mouth disease (FMD) is a highly contagious and economically important disease caused by foot-and-mouth disease virus (FMDV). Animals that can be affected include cattle, buffaloes, sheep, goats, pigs and wild ruminants [1]. FMDV is a positive sense, single-stranded RNA virus (genus *Aphthovirus*, family *Picornaviridae*) occurring in seven serotypes, O, A, C, Asia 1, SAT 1, SAT 2 and SAT 3, each with a wide spectrum of antigenic and epidemiological distinct subtypes. The wide diversity is considered a consequence of the high mutation rate, quasi-species dynamics and recombination [2,3].

FMD is endemic in Pakistan [4] and causes huge economic losses to commercial cattle and buffalo owners. According to the Food and Agriculture Organization of the United Nations (FAO) there are no proper arrangements for providing vaccine to the farmers and the open market is flooded with uncontrolled vaccine of doubtful efficiency [5].

FMD is considered endemic with the serotypes O, A and Asia 1 in both Pakistan [6] and the neighbouring countries of India, Afghanistan, Iran and China [7-9] and those serotypes are a continued problem in Pakistan.

According to the OIE HandiSTATUS [10] Pakistan considers itself as having a seasonal, low-level, sporadic occurrence of FMD (Pakistan reported around 10–30 outbreaks per year until year 2000 after which no information is available). Animals are only vaccinated upon request and the yearly number of vaccine doses used varies between 12,000 to 95,000 doses for cattle and 7,000 to 60,000 for buffaloes in the years from 1997–2002 (no data available after 2002) [10]. This amount of vaccine is likely in addition to an unknown amount of open market, uncontrolled vaccines, but is nevertheless not much considering that Pakistan has a population of 51,1 million cattle, 56,9 million buffaloes, 50,3 million sheep and 123,9 million goats [4].

The majority of commercial dairy farmers are vaccinating their animals against FMD, either with imported trivalent vaccine, e.g. Aftovax (Merial, France), or with a locally produced monovalent vaccine (serotype O) [6].

Major challenges to control FMD in Pakistan relate, in part, to the lack of sufficient resources for diagnosis and continuous FMD genotype surveillance, but also the difficulties of controlling the vaccine market, as well as the lack of basic biosecurity awareness and control of animal movements. The latter is also hampered by the annual religious festival Eid ul-Azza, where thousands of buffaloes, cattle and small ruminants are transported across the country.

The present work focuses on the Landhi Dairy Colony (LDC), located in the suburbs of Karachi in the Sindh province of South-Pakistan. LDC is the largest dairy colony in Pakistan and the largest Buffalo colony in the world. It was established in 1959 within an area of 752 acres (incl. 250 acres for roads, shops and other facilities) for 15,000 animals, but there are now more than 300,000 dairy animals (> 95% buffaloes) on approximately 2000 farms and an unknown number of sheep and goats, which are freely running around in the whole colony. This overload, and unclear land ownership leads to hygiene and environmental problems. The majority of the milking animals in LDC are kept only for one lactation phase and consequently approximately 10–12% of the population is replaced every month.

After the lactation period the majority of the animals are sold to breeders or for slaughter and only a few are kept by the dairy farmers for re-breeding. Most of the animals are brought to and from the animal rich districts of Punjab and Sindh provinces.

Previous studies employing participatory epidemiology indicated a relatively high annually FMD prevalence between 41% and 50% in the southern Sindh region around Karachi [6].

To develop an effective vaccination strategy it is crucial to understand the dynamic of the disease and thereby indicating the best time points of administering the vaccine. Thus, individually vaccination is already performed on the large ruminant population, but with vaccines of variable quality and efficiency, it is likely that the majority of potential FMDV infections are subclinical and therefore not recognised. From April 2006 to April 2007 we collected monthly a number of mouth-swabs from apparently healthy buffaloes and cattle, applying a convenient sampling scheme based on a two-stage random sampling setup, in conjunction with participatory information from each selected farm. The total number of collected samples was 960 mouth-swabs from 124 farms.

Furthermore, we collected epithelium samples from clinically affected animals as well as mouth-swab samples from farms with a recent FMD outbreak, and 180 serum samples collected from slaughtered animals in the period from October 2006 to March 2007. The collection of probang and blood samples from living cattle or buffaloes was considered not possible due to socio-religious reasons.

Samples have been screened for FMDV by real-time RT-PCR [11,12] and the partial 1D coding region of selected, FMDV positive isolates, has been sequenced. In addition, the full 1D coding region of a locally produced monovalent vaccine (serotype O) has been sequenced to examine the relatedness of vaccine strain to the circulating serotype O lineages. Serum samples have been analysed by applying serotype O, A and Asia 1 specific antibody ELISA [13] and non-structural proteins (NSP) ELISA [14].

This work will help to develop an appropriate vaccination strategy for Pakistan's largest dairy colony, including the choice of the best matching vaccines, as well as helping to improve our understanding of the epidemiology of FMD.

## Results

### Infection prevalence

We randomly selected farms in LDC and took swab samples from randomly selected animals for a subsequently screening for FMDV genome by real-time RT-PCR. We aimed to get information from farms where no animals with clinical signs of FMD were present, judged by personal examination or by examination done by the local veterinarians and information from the respective farmer. If there has been at least one animal showing either acute FMD or healing FMD lesions, we excluded those farms from the FMDV infection prevalence analysis at aggregate level and calculated the within-farm prevalence separately for detecting potential FMDV prevalence differences.

Table 1 shows the prevalence of each FMDV infection-positive farm, without any signs of clinical FMD, per month in relation to the farm population. Confidence intervals were calculated for a normal distributed population without finite population correction factor. This means that some confidence intervals related to a very small sample size or extreme point estimates are doubtful (shown in grey in Table 1). However, we believe that the shown point estimates, i.e. prevalence values, are reliable and that the shown confidence intervals give useful, additional information. The mean prevalence for those farms with PCR-positive animals that were randomly selected and without animals showing clinical signs of FMD, is 19.2% (SE 3.99%). Table 2 shows the prevalence for each infection positive found farm per month on which during the sampling, animals with healing FMD lesions were detected. The mean prevalence here is 53.9% (SE 15.08%). Applying t-test statistics demonstrate that the mean prevalence in the latter group was significant higher than in the farms where no animals with healing lesions were detected. The t-statistic for H_1 _(mean prevalence on farms with animals with healing FMD lesions > prevalence on farms without animals with healing FMD lesions) at the 0.05 critical alpha level, t(22) = 3.17, p= 0.0022. For farms with ongoing FMD, i.e. at least one animal show signs of acute FMD, a mean prevalence of 87% could be detected (Table 3). As swab samples for the latter were only collected in April 2006 from two farms with acute FMD, the sample size was considered to be too low to allow a meaningful statistical analysis. However, the FMDV prevalence in these two farms appeared higher than in the farms containing animals with healing lesions.

**Table 1:** Prevalence for each FMDV infection positive found farm per month in relation to the farm population

**Month **[total number of farms sampled]	**Farm ID.**	**total farm Population**	**sampled**	**infected**	**Prevalence**	**l. CI**	**u. CI**
April 2006 [18]	3	1500	13	1	8%	0%	22%
	7	-	9	6	67%	36%	97%
	8	250	9	1	11%	0%	32%
	11	360	9	1	11%	0%	32%
	15	200	9	1	11%	0%	32%

May 2006 [7]	3	193	6	6	100%	100%	100%

August 2006 [9]	1	197	3	3	100%	100%	100%
	2	131	3	1	33%	0%	87%
	3	46	3	3	100%	100%	100%
	4	140	3	2	67%	13%	100%
	5	143	3	2	67%	13%	100%
	6	370	3	1	33%	0%	87%
	7	58	3	1	33%	0%	87%
	8	55	3	1	33%	0%	87%
	9	63	3	3	100%	100%	100%

September 2006 [19]	5	145	6	4	67%	29%	104%
	6	145	12	1	8%	0%	24%
	7	110	9	2	22%	0%	49%
	10	190	9	1	11%	0%	32%
	12	70	9	1	11%	0%	32%

October 2006 [5]	3	150	5	1	20%	0%	55%

November 2006 [5]	5	260	6	1	17%	0%	46%

December 2006 [5]	2	-	5	1	20%	0%	55%
	3	700	6	6	100%	100%	100%
	4	90	6	4	67%	29%	100%
	5	196	5	3	60%	17%	100%

January 2007 [17]	2	120	10	2	20%	0%	45%
	7	107	9	1	11%	0%	32%
	8	143	20	8	40%	19%	61%
	9	272	10	1	10%	0%	29%
	11	111	9	1	11%	0%	32%
	13	214	10	1	10%	0%	29%
	17	505	9	1	11%	0%	32%

February 2007 [5]	1	266	6	1	17%	0%	46%
	2	162	6	5	83%	54%	100%
	3	124	6	4	67%	29%	100%
	4	372	6	2	33%	0%	71%
	5	266	6	5	83%	54%	100%

March 2007 [5]	1	150	6	5	83%	54%	100%
	2	175	6	3	50%	10%	90%
	3	65	6	2	33%	0%	71%
	4	80	6	2	33%	0%	71%
	5	122	6	3	50%	10%	90%

April 2007 [17]	1	205	9	1	11%	0%	32%
	12	145	9	2	22%	0%	49%
	15	122	9	1	11%	0%	32%

Confidence intervals were calculated for a normal distributed population without finite population correction factor. This means that some confidence intervals related to very small sample size or extreme point estimates are doubtful (shown in grey).

**Table 2:** Prevalence for each infection positive found farm per month on which during the sampling, animals with healing FMD lesions were detected

**Month**	**Farm ID.**	**total farm Population**	**sampled**	**infected**	**Prevalence**	**l. CI**	**u. CI**
April 2006	21	180	9	8	89%	68%	100%
March 2006	22	120	10	2	20%	0%	45%
September 2006	5a	145	6	4	67%	29%	100%
January 2007	17	-	20	8	40%	19%	61%

**Table 3:** Prevalence for farms with ongoing FMD

**Month**	**Farm ID.**	**total farm Population**	**sampled**	**infected**	**Prevalence**	**l. CI**	**u. CI**
April 2006	3A	269	9	8	89%	68%	100%
April 2006	7A	121	20	17	85%	69%	100%

**Figure 1:**
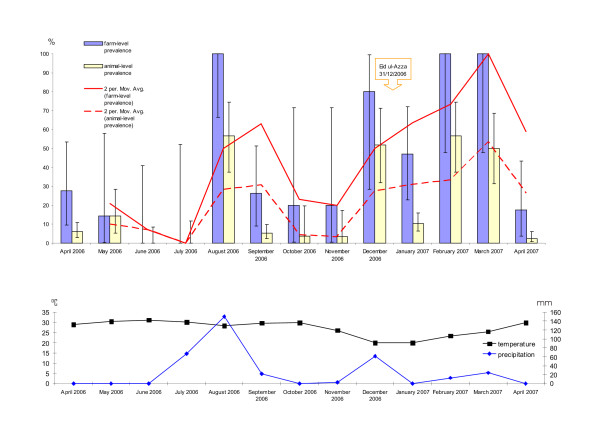
**FMDV infection prevalence at aggregate level**. The farm-level (herd-level) prevalence reflects the number of farms with FMDV infection positive found animals and the animal-level prevalence reflect the number of FMDV infection positive found animals within the sampled population. Both prevalence values are shown with the exact binomial confidence interval. Furthermore, the moving average (SMA) for both measures is displayed and the date of Eid ul-Azza is indicated. In the lower panel temperature and precipitation measured in Karachi in the period of April 2006 to April 2007 is displayed.

Figure 1 displays the FMDV infection prevalence at aggregate level from April 2006 to April 2007, based on the number of inapparently infected animals found in a two-stage sampling scheme. The farm-level (herd-level) prevalence reflects the number of farms with positive animals, calculated as the proportion of Σ farms with infected animals per month to Σ farms sampled per month, and the animal-level prevalence reflect the number of FMDV positive found animals within the sampled population, calculated as the proportion of Σ animals infected per month to Σ animals sampled per month (see also additional file 1). Both prevalence values are shown with the exact binomial confidence interval, a method using the cumulative probabilities of the binomial distribution and therewith expressing the situation in the whole LDC. Both measures show an endemic, frequent occurrence of FMD in the colony, with peaks in August 2006, December 2006 and February 2007 to March 2007. In conformity with the prevalence, the precipitation peaks in August, December, February and March. Applying the Pearson-correlation statistics for animal-level prevalence to precipitation demonstrates a significant association, with a correlation coefficient ρ = 0.57 and the t-statistic for H_1 _(ρ > 0) at the 0.05 critical alpha level, t(11) = 2.27, p= 0.021.

Moreover, the moving average analysis (Figure 1), which removes random variations within the point estimates, show an appreciable increase from December 2006 to March 2007, expressing the cumulative effect of the second rain season, the Eid ul-Azza festival and possibly the slightly cooler temperature during this period. The temperature in Karachi between April 2006 and April 2007 ranged between 20°C and 30,5°C.

### Participatory information

During sampling the owners of the farms have been interviewed with regard to their FMD vaccine practice. Table 4 shows that 88% of all questioned farmers vaccinated their animals. Of those, 79% were using the trivalent Aftovax-vaccine (Merial, France) and 9% the local monovalent (serotype O) vaccine. Four percent of the farmers were vaccinating their animals on regular basis twice a year, whereas the majority of the farmers vaccinated only the new entrants to the farm. All interviewed farmers, which vaccinated their animals, administered the vaccine only once and not as recommend with an additional booster vaccination two to six weeks after the initial vaccination.

**Table 4:** Vaccine use on all questioned farms

No. Farms	vaccinating	local mono-valent (O) vaccine	Aftovax^©^	other vaccine	unknown	not vaccinating
127	112	11	101	1	4	10
**Percent →**	**88**	**9**	**79**	**1**	**3**	**8**

The second line represents percent either in relation to the number of total questioned farms (vaccinating = 88%) or to the number of vaccinating farms.

### Sero-surveillance

**Figure 2:**
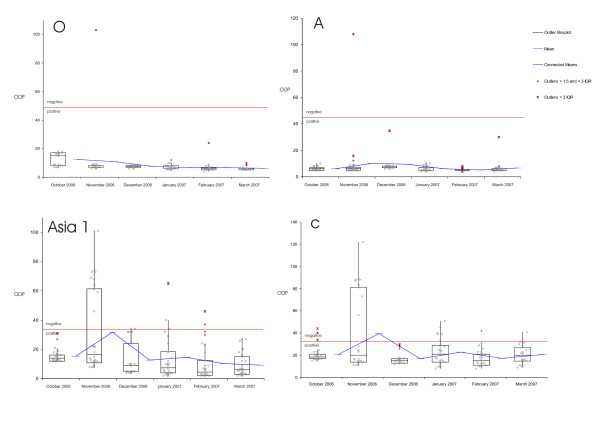
**Descriptive statistics of the antibody ELISA for samples per month and serotype**. Box-and-whisker diagram of the measured optical density percent (ODP) per month and serotype. Showing the smallest observation, lower quartile (Q1), median, upper quartile (Q3), and largest observation. In addition outliers according their interquartile range (IQR) and means are displayed. Each circle represents the measured ODP of a sample. The red line represents the threshold for each serotype, i.e. samples are considered negative if the ODP is for O >= 50, for A >= 45, for Asia 1 >= 35 and for C >= 35.

From October 2006 to March 2007 we collected monthly serum samples from 30 randomly selected Asian Buffaloes in LDC at the local slaughter house, immediately after the death of the animals. Figure 2 shows the results of the antibody ELISA for those 180 samples per month and serotype. The data for serotype O shows a high amount of antibodies (low ODP) for the whole period of time, with a small variance of measured values. The same is true for serotype A.

**Figure 3:**
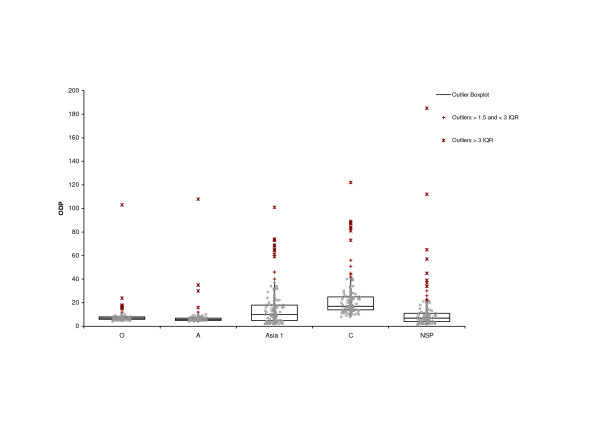
**Descriptive statistics of the ELISA results for all samples at a serum dilution of 1/5**. Box-and-whisker diagram of the measured optical density percent (ODP). Showing the smallest observation, lower quartile (Q1), median, upper quartile (Q3), and largest observation. In addition outliers according their interquartile range (IQR) are displayed. Each circle represents the measured ODP of one sample.

In our analysis antibodies against serotypes Asia 1 and C, show generally a higher variance per month than those against the other serotypes, but the Median for each month is clearly positive (Figure 2). All 180 samples have been the tested for antibodies against the non-structural proteins of FMDV and all but four have been found positive (Figure 3 and additional file 2).

Figure 3 shows the distribution of all 180 collected serum samples per serotype at a serum dilution of 1/5. A high antibody response (ODP < 10) can be seen for serotypes A and O and against the non-structural proteins (NSP). The median for the antibody response against Asia 1 has an ODP value of 12 and against serotype C of 18 respectively.

**Figure 4:**
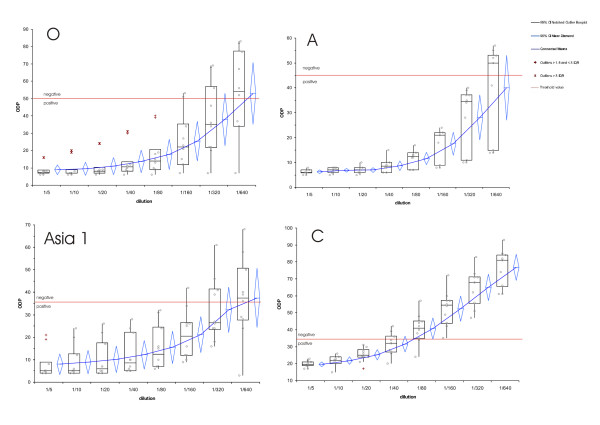
**Descriptive statistics of the antibody ELISA for 10 randomly selected samples per serum-dilution and serotype**. Box-and-whisker diagram of the measured optical density percent (ODP) per dilution and serotype. Showing the smallest observation, lower quartile (Q1), median, upper quartile (Q3), and largest observation. In addition outliers according their interquartile range (IQR) and means are displayed. The top and bottom diamond vertices are the respective upper and lower 95% confidence limits (CI) about the group mean. Each circle represents the measured ODP of a sample. The red line represents the threshold for each serotype, i.e samples are considered negative if the ODP is for O >= 50, for A >= 45, for Asia 1 >= 35 and for C >= 35.

We randomly selected ten serum samples to determine the highest serum dilution that gives a positive signal in ELISA for each serotype (Figure 4). The Median for all tested serotypes, except for serotype C, is positive with a serum dilution of 1/320. Some tested sera are still positive at a dilution 1/640 and above. The highest serum dilution that gives a positive signal for serotype C is 1/40 (Median). The calculated ODP means for the serotypes O, A and Asia1 are at a 1/5 serum-dilution 9 (σ = 4), 6 (σ = 1), 8 (σ = 6), and those result in an endpoint-titre of 1/320, with a standard deviation of one twofold dilution step. For serotype C the calculated ODP mean at a 1/5 serum-dilution is 20 (σ = 2), resulting in an endpoint-titre of 1/40, with a standard deviation of one dilution step.

**Figure 5:**
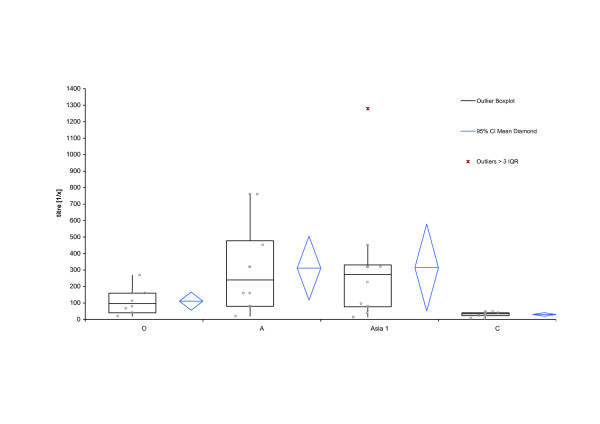
**Descriptive statistics of the virus neutralisation test for 10 randomly selected samples per serotype**. Box-and-whisker diagram of the calculated titres for each serotype. Showing the smallest observation, lower quartile (Q1), median, upper quartile (Q3), and largest observation. In addition outliers according their interquartile range (IQR) and means are displayed. The top and bottom diamond vertices are the respective upper and lower 95% confidence limits (CI) about the group mean. Each circle represents the calculated titre of a sample.

Furthermore we determined for those ten selected serum samples the endpoint-titre in virus neutralisation for each serotype (Figure 5). Generally; the virus neutralisation titres are consistent with the results of the ELISA titration. The Median for all tested serotypes, except for serotype C, has an endpoint-titre of equal or above 1/100. VNT analysis for serotype O isolates displays a relative small variance with a Median of approximately 1/100. Serotype A isolates display the highest variance, but with a Median of 1/260. For Asia 1 isolates the Median is 1/280, displaying a medium variance compared to the others, and for C 1/50, with a very small variance.

### Phylogenetic analysis

Out of the 106 FMDV positive swab-samples from animals with and without clinical signs it was possible to sequence the partial or full 1D coding region, which encodes for the immuno-dominant VP1 surface protein, from 58 samples. In addition we sequenced the full 1D genome region of 17 epithelium samples collected during 2006, mainly from LDC, but also some from outside LDC and from farms around Islamabad. From all sequenced samples, 19 belong to serotype O, hereof ten epithelium samples, and 56 to serotype A, hereof seven epithelium samples.

**Figure 6:**
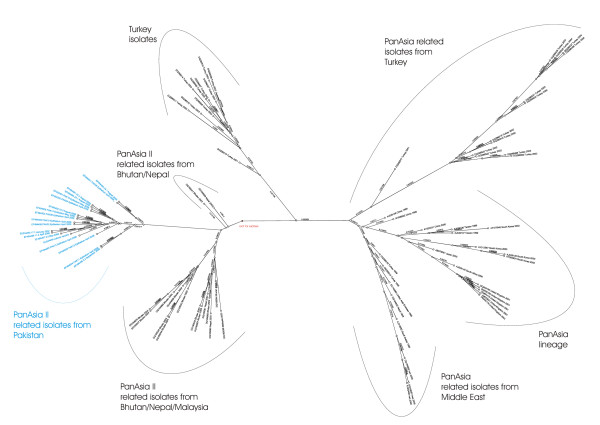
**Unrooted phylogenetic tree of the partial 1D (VP1) nucleotide sequence of Pakistani serotype O isolates and related published sequences**. The root for subtree (Figure 5) is indicated.

**Figure 7:**
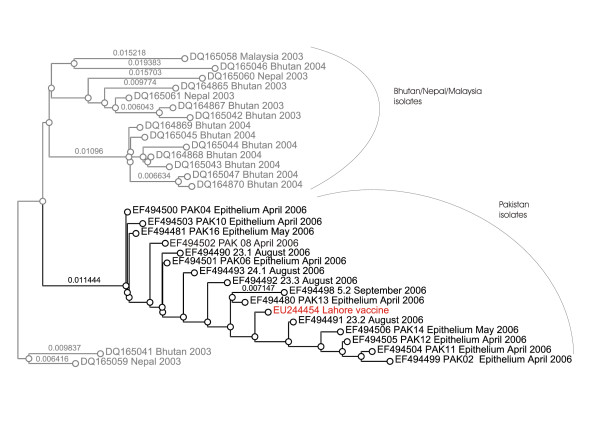
**Bayesian phylogenetic analysis of the full 1D (VP1) nucleotide sequence of Pakistan serotype O isolates (black) and closely related published sequences (grey)**. The local produced monovalent vaccine (Lahore vaccine) is indicated in red.

**Figure 8:**
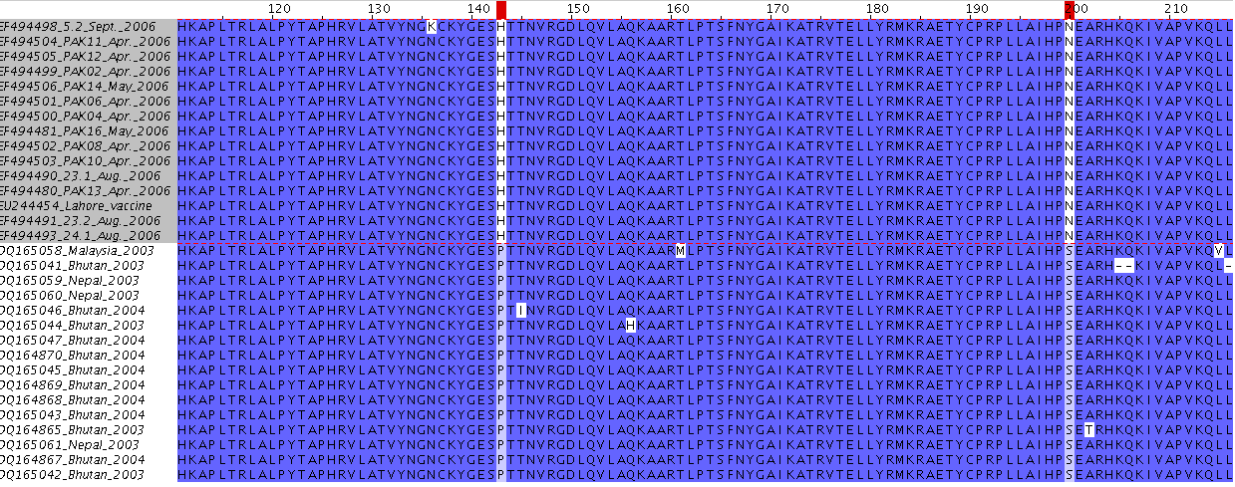
Deduced amino acid sequence of the partial VP1 sequence of the serotype O isolates and related sequences from Malaysia, Bhutan and Nepal.

Figure 6 shows the unrooted phylogenetic tree of the Pakistani serotype O isolates in relation to similar serotype O sequences, published in Genbank. The serotype O isolates from the Pakistan cluster are monophyletic, i.e. share a common ancestor. The most related isolates originate from Bhutan/Nepal, collected between 2003 and 2004. The latter belong to a new PanAsia lineage described by the OIE/FAO World Reference Laboratory for Foot-and-Mouth Disease in 2007 and designated PanAsia II [15]. Figure 7 shows a subtree of serotype O, containing only sequences from Pakistan, Bhutan, Nepal and one from Malaysia. This phylogram shows the close relationship between the isolates from Bhutan/Nepal and Pakistan. Noticing the small branch lengths, it is remarkably that the sequence derived from the local-monovalent O vaccine is placed in very close relation to samples derived from infected animals. Figure 8 displays the deduced amino acid sequence of the partial VP1 sequence of the serotype O isolates and related sequences from Malaysia, Bhutan and Nepal. There is a very high amino acid conservation between those isolates, even as they are collected during a time range from 2003 to 2006. However, the Pakistan isolates are clearly distinct to the isolates from Malaysia, Bhutan and Nepal at residues 143 and 200. Residue 143, located four amino acids before the RGD motif in the GH-loop, in the Pakistan isolates contain a histidine, whereas the others, similar to the majority of other published serotype O sequences, have a proline at this position; thus proline has a cyclic ring and its presence creates a fixed kink in a protein chain, its presence lead to a change in the secondary structure. Furthermore, residue 200 in the isolates from Pakistan contains asparagine instead of serine, as the majority of other published serotype O sequences.

**Figure 9:**
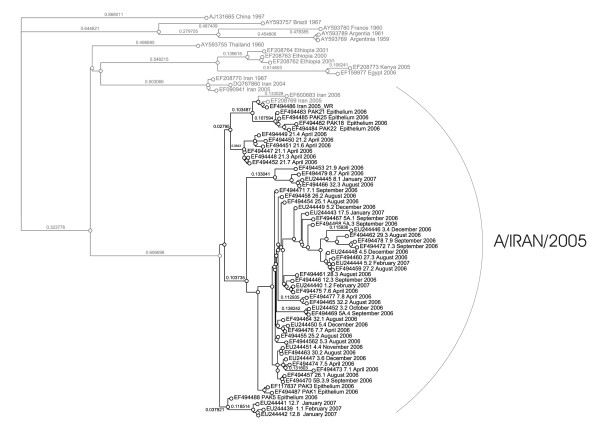
Bayesian phylogenetic analysis of the partial 1D (VP1) nucleotide sequence of Pakistan serotype A isolates (black) and closely related published sequences (grey).

Figure 9 shows the phylogram of the serotype A isolates. All Pakistani isolates belong to the recent discovered A/Iran/2005 lineage. The branch lengths here are, typically for serotype A, larger than those of serotype O.

### Virulence and host species

It has been shown previously that of those animals in this study infected with the FMDV A/Iran/2005 lineage, the majority of clinically affected animals are cattle [16]. Regarding the FMDV type O infected animals; six of ten epithelium samples from clinically affected animals are from buffaloes and only one of seven subclinically infected animals originate from cattle. This displays the LDC population of more then 95% Asian Buffaloes and indicates an equal distribution of serotype O caused clinical FMD between bovine and buffalo species. In contrast to the A/Iran/2005 lineage, where the occurrence of clinical FMD seems to be host species dependent, is there no indication of host species dependence in the serotype O caused outbreaks.

## Discussion

Landhi Dairy Colony contains a relatively high proportion of vaccinated cattle and buffaloes (Table 4). However, the vaccination is mainly performed once and mainly on newly introduced animals. Within such a population a high FMDV challenge, with the vaccine covered sero/sub-type, against animals with a high immunity or a low challenge in animals with low vaccine titres, may both produce subclinical disease [12,17]. During our study we have seen only a sporadic occurrence of animals with clinical signs of FMD, mainly in April 2006 and mainly in cattle. The latter may be explained by our findings that the majority of FMDV infections were caused by the A/Iran/2005 lineage, which seems to cause mainly subclinical disease in buffaloes [16] and thereby possibly outplay the serotype O FMDV, but also through the relatively better efficiency of the applied vaccines towards serotype O. Nevertheless, we have detected an endemic FMDV infection occurrence (Figure 1), i.e. an endemic occurrence of mainly subclinical FMD, peaking in August 2006 and December 2006 to March 2007. The maxima in August and December are in clear correlation to the measured precipitation and consequently with the increased humidity during the rainy periods. The relationship between humidity and virus transmission/stability has been described in several publications [18-21]. The prevalence peaks in February and March 2007 can be explained by the cumulative effect of humidity, cooler temperature and the introduction of new animals, potentially FMD infected, from all over the country during the Eid ul-Azza festival. Assuming that the incubation period of FMD in Asian Buffaloes is similar to that in cattle, i.e. 2 to 14 days [1,22] the spread of FMDV to the whole colony in February and March is likely, in particular considering the intensive movement of animals and the lack of biosecurity awareness.

Given that the detection window for FMDV in mouth swabs by real-time RT-PCR is approximately 14 days [12] and that our results indicate a FMDV infection mean prevalence of 19,2% per month (Table 1), a yearly FMDV incidence proportion of approximately 458% (calculated as incidence proportion = prevalence/duration) can be assumed, which means that there is a high risk that a very large proportion, if not all, animals in LDC become infected with FMDV during the period of one year. Id est, there is continuous FMDV circulation in LDC. This FMDV maintenance in LDC bear also a risk of FMDV spreading to other parts of Pakistan, hence animals that leave the colony, e.g. for re-breeding, can be infected and transmit the disease to other animal populations.

The serological analysis shows that 176 of 180 serologically tested animals are positive in NSP ELISA and the majority of those animals have been confronted with structural antigens from all present serotypes. However, this does not necessarily mean that they have acquired immunity by becoming infected with each serotype. We consider it more likely that those animals have been vaccinated with multivalent vaccines, either after they have had an infection or the vaccine strain has not matched with the circulating strain. The relatively low titres for serotype C support this consideration, since only a minority of available vaccines contain serotype C antigens. Even if it is possible that they have been vaccinated with a not properly inactivated or purified vaccine, does the relative strong signals for the NSP ELISA (Figure 3) not support this, assuming that there is some form of a NSP purification step included in the vaccine production, even in the black market vaccines.

Figure 5 shows that the calculated median endpoint-titre, in the virus neutralisation assay, for all tested serotypes, except for serotype C, is equal or above 1/100 and thus a good protection status of the tested animals against the serotypes O, A and Asia 1 can be assumed. The relatively low endpoint-titre of 1/50 for serotype C may indicate that vaccines containing this very seldom serotype are still in use in Pakistan, but not as frequently administered to the animals as vaccines for the other serotypes and likely not recently boosted by circulating serotype C FMDV. Compared with the ELISA titration (Figure 4), were the median endpoint-titres of the serotypes O, A and Asia 1 are equal at 1/320, is the endpoint-titre for serotype O lower in the virus neutralisation assay. This can be explained by the fact that both methods are performed with the O Manisa lineage and that the ELISA is more robust against virus lineage variations. Also, as we do not have any further data on those serologically tested animals it can be assumed that the average age of the slaughtered buffaloes is between 4 and 7 years, based on the information from farms were we collected mouth swab samples. Furthermore, due to the tradition of keeping animals only for one lactation period and purchasing them from other areas of Pakistan, this result represent more the FMD situation of whole Pakistan than the particular situation in LDC, thus the time point of infection can have been before the animal was brought to LDC.

The phylogenetic analysis of the 65% of the positive samples sequenced shows that primarily two virus-lineages have circulated in LDC from April 2006 to April 2007. Namely, A/Iran/2005, a serotype A lineage which caused major outbreaks in cattle in Turkey, Egypt and Jordan during 2006 and 2007, and a Pakistan specific serotype O lineage. The A/Iran/2005 lineage is extensively described elsewhere [16]. The serotype O sequences constitute a monophyletic group, not related to the "old" PanAsia lineage, but related to the recently described PanAsia II lineage [15]. Thus, vaccine strains covering the PanAsia lineage of serotype O, e.g. O Manisa-like vaccines, are not necessary giving a good protection to this lineage. The latter is also supported by the differences of the median endpoint-titres for serotype O in ELISA and serum neutralisation assay. Consequently, it can be argued that those vaccines will lose their efficiency, after further FMDV evolution, away from the "old" PanAsia lineage. The most related sequences to the Pakistani originate, with the exception of one sequence from Malaysia, from Nepal and Bhutan collected during 2003 and 2004. A common history of the Pakistan and Bhutan/Nepal lineage can not be excluded, but the fixed differences at residues 143 and 200 of the VP1 protein indicates that each has established its own ecological niche.

Remarkably, the sequence of the locally produced monovalent-O vaccine (Lahore vaccine) is nearly identical with the sequences of the field strains. According to representatives of the vaccine company in Lahore, the company has been using the same vaccine strain for approximately 30 years. However, it is the opinion of the authors that this appears highly unlikely, due to the striking similarity of the 1D vaccine sequence to the field samples sequence. Probably, there has been an unmeant contamination of the vaccine production unit or the information we received from the company is not correct.

## Conclusion

For an effective and realisable FMD control program in LDC, we suggest to introduce a twice annually mass vaccination of all buffaloes and cattle in the colony. These mass vaccinations should optimally take place shortly before the beginning of the two rainy periods, e.g. in June and September. Those vaccinations should, in our opinion, be in addition to the already individually performed vaccinations of single animals, as the latter usually targets only newly introduced animals. This suggested combination of mass vaccination of all large ruminants with the already performed individually vaccination should provide a continuous high level of herd immunity in the entire colony.

Vaccines used for this purpose should contain the matching vaccine strains, i.e. A/Iran/2005 and the regional type of serotype O (PanAsia II), but also antigens of the, in this world region endemic, Asia 1 lineage should be included. As alternative for A/Iran/2005, a vaccine containing the A22 lineage could potentially be used [23]. For covering the O sublineage, the locally produced monovalent vaccine (Lahore vaccine) could be used, if it is assured that this vaccine is similar to the one we purchased and that it is properly inactivated, purified and with a sufficiently high antigen content. It is important that a continuous FMDV surveillance, including sublineage typing, is carried out, to identify potentially newly introduced FMDV lineages and therewith subsequently to be enable good advice on the choice of the best vaccine strains.

In the long term it will be important to control the vaccine use, so that subclinical FMD will be avoided.

## Methods

### Sampling scheme and sample handling

A repeated cross-sectional survey has been performed by collecting a minimum of 162 mouth swabs from randomly selected animals in selected farms (9 animals from each of 18 farms) four times, during April and September 2006 and January and April 2007, combined with a monthly sampling of approximately 30 swabs (5 animals from each of 6 farms). The survey was based on a 2 stage cluster-randomised setup, with farms as first unit of randomisation and animals on the farms as second unit. To confirm an geographical randomised sampling we indexed each selected farm by latitude and longitude with a GPS device and plotted the coordinates, using GoogleEarth, on a map of LDC.

Mouth swabs have been taken from apparently healthy animals. If during sampling, animals with healing lesions were detected, those farms were excluded from prevalence calculations for aggregate level prevalence (Figure 1).

In addition, we purchased a bottle containing the monovalent local produced type O vaccine in a drug store in LDC.

Plain Plastic/Rayon swabs (Sterilin^®^, U.K.) in a sterile tube have been used for mouth swab collection. The swab sample was taken by carefully holding the animal with the mouth slightly open and than moving the swab up and down on the surface of the tongue four to five times. The tip of the swabs was than stored in a 2 ml tube containing 1 ml RLT-buffer (Qiagen, Germany), to preserve any viral RNA present.

In addition, we collected epithelium samples from clinically affected animals. These animals were not randomly selected. The tongue epithelium was collected from unruptured or freshly ruptured vesicles by gently abrading it with a glove, with rubber dots, grabbing the tongue with the gloved hand and pulling along the surface.

Epithelium was than placed in vials containing buffered glycerol (50% glycerol with 50% phosphate buffer, pH 7,6), and kept initially at 4°C and subsequently at -20°C.

All samples were, in compliance with the applicable regulations, sent to National Veterinary Institute, Technical University of Denmark, Lindholm, for further analysis.

### RT-PCR, sequencing and phylogenetic analysis

Total RNA of all collected swab samples was extracted using QIAamp RNA Blood Kit (Qiagen, Hilden, Germany) according to the manufacturer's instructions. The Real-Time RT-PCR described by [11] was used to screen the samples for FMDV RNA.

Tissue (50–100 mg) was homogenized in 1 ml RNA*pro*™ Solution (Qbiogene, USA) in a Lysing Matrix D tube (Qbiogene) using a FP 120 Fast Prep™ Cell Disruptor (Qbiogene). Total RNA was extracted using RNeasy-Mini Kit™ (Qiagen) according to the manufacturer's instructions.

cDNA synthesis for tissue samples and positive swab samples was done using Ready-To-Go™ You-Prime First-Strand Beads (GE Healthcare Life Sciences, Sweden), employing the primers NV27T (IUPAC code) and random hexamers pdN6 (IUPAC code).

Five μl of the template cDNA were added to 45 μl of the PCR reaction mixture containing 0,2 μM primers, 200 μM each of dATP, dCTP, dGTP and dTTP, 10 mM Tris-HCl (pH 8.3), 50 mM KCl, 1.5 mM MgCl2 and 1 U of AmpliTaq^® ^Gold DNA polymerase (Applied Biosystems, UK). DNA was amplified with a DNA Thermal Cycler PE9700 (Perkin Elmer) by a two-step cycling reaction as follows: 95°C for 15 min, and five cycles of 94°C for 30 sec, 57°C for 2 min and 72°C for 30 sec, and then 35 cycles of 94°C for 30 sec, 61°C for 30 sec and 72°C for 30 sec, followed by a final extension step of 72°C for 10 min. PCR primers used have been described elsewhere [16,24].

The resulting PCR products were examined by electrophoresis, using a 1,2% agarose gel, with a separation time of 1.5 hours at 6.5 V/cm. Amplicons were visualised with ethidium bromide and subsequently extracted and purified from the agarose gel with QIAquick Gel Extraction kit (Qiagen). Cycle-sequencing, using PCR primers, was then performed by Agowa GmbH, Germany.

Sequence assembling was performed with ContigExpress (VectorNTI^©^-software) and multiple alignment was performed by log-expectation comparison, using the MUSCLE (v.3.6) software [25].

Models of evolution were determined by hierarchical Likelihood-Ratio test of 24 substitution models, using the programs PAUP*(v. 10) (Sinnauer, U.K.) and MrModeltest (v. 2.2) [26].

For serotype A the Hasegawa-Kishino-Yano plus Gamma (HKY+G) model was used and Bayesian analysis was performed using MrBayes (v3.2) [27] with the following settings. The maximum likelihood model employed 2 substitution types ("nst = 2"), with base frequencies set to fixed values ("statefreqpr = fixed"). Rate variation across sites was modelled using a gamma distribution (rates = "gamma"). The Markov chain Monte Carlo search was run with 4 chains for 500000 generations, with trees begin sampled every 100 generations (the first 1000 trees were discarded as "burnin").

For serotype O the General Time Reversible plus Gamma (GTR+G) model was used and Bayesian analysis was performed using MrBayes (v3.2) [27] with the following settings. The maximum likelihood model employed 6 substitution types ("nst = 6"), with base frequencies set to variable values ("statefreqpr = dirichlet(1,1,1,1)"). Rate variation across sites was modelled using a gamma distribution (rates = "invgamma"). The Markov chain Monte Carlo search was run with 4 chains for 500000 generations, with trees begin sampled every 100 generations (the first 1000 trees were discarded as "burnin").

### ELISA

For the detection of antibodies against FMDV in serum, a blocking ELISA assay was carried out as described by Have and Jensen [13] using O-Manisa, A22-Iraq, Asia 1-Shamir and C-Noville antigens. For detecting antibodies against non-structural proteins, a blocking ELISA assay was carried out as described by Sorensen et al. [14].

### Virus neutralisation test (VNT)

VNT was performed according to OIÈ's Manual of Diagnostic Tests and Vaccines for Terrestrial Animals 2007 [28] and titres calculated according to Kärber [29].

### Statistical analysis

Statistical analysis was performed using R [30] and MS-Excel.

### Climate data

Climate data for Karachi were obtained from a database of Germany's National Meteorological Service, the Deutscher Wetterdienst [31].

## Authors' contributions

JK participated in planning of the study and carried out the molecular and field epidemiological analysis, participated in the field work and drafted the manuscript. MH and MA participated in the field work and delivered background information. SA was project coordinator and conceived the study and helped in the field work and to draft the manuscript. All authors read and approved the final manuscript.

## Supplementary Material

Additional file 1 FMDV infection prevalence at aggregate level in tabular form. FMDV infection prevalence at aggregate level from April 2006 to April 2007, based on the number of inapparently infected animals found in a two-stage sampling scheme. The farm-level (herd-level) prevalence reflects the number of farms with positive found animals, calculated as the proportion of Σ farms infected per month to Σ farms sampled per month, and the animal-level prevalence reflect the number of positive animals within the sampled population, calculated as the proportion of Σ animals infected per month to Σ animals sampled per month Click here for file

Additional file 2 ODP values of all 180 tested serum samples. ODP values of all 180 tested serum samples, for serotype specific and NSP ELISA Click here for file
