# Supplementary material for: Epidemiology of foot-and-mouth disease in Landhi Dairy Colony, Pakistan, the world largest Buffalo colony
Source: Virol J. 2008 Apr 29;5:53. doi: 10.1186/1743-422X-5-53 (PMC2386124; doi:10.1186/1743-422X-5-53)
Supplement: Additional file 1 — FMDV infection prevalence at aggregate level in tabular form. FMDV infection prevalence at aggregate level from April 2006 to April 2007, based on the number of inapparently infected animals found in a two-stage sampling scheme. The farm-level (herd-level) prevalence reflects the number of farms with positive found animals, calculated as the proportion of Σ farms infected per month to Σ farms sampled per month, and the animal-level prevalence reflect the number of positive animals within the sampled population, calculated as the proportion of Σ animals infected per month to Σ animals sampled per month [file 1743-422X-5-53-S1.pdf]

Month	No. farms	No. positive farms	farm-level prevalence	l.level	u.level
**April 2006**	18	5	28	10	53
**May 2006**	7	1	14	0	58
**June 2006**	7	0	0	0	41
**July 2006**	5	0	0	0	52
**August 2006**	9	9	100	66	100
**September 2006**	19	5	26	9	51
**October 2006**	5	1	20	1	72
**November 2006**	5	1	20	1	72
**December 2006**	5	4	80	28	99
**January 2007**	17	8	47	23	72
**February 2007**	5	5	100	48	100
**March 2007**	5	5	100	48	100
**April 2007**	17	3	18	4	43

animal level

Month	No. animals sample	No. infected animals	Animal-level prevalence	l.level	u.level
**April 2006**	166	10	6	3	11
**May 2006**	42	6	14	5	29
**June 2006**	42	0	0	0	8
**July 2006**	30	0	0	0	12
**August 2006**	30	17	57	37	75
**September 2006**	171	9	5	2	10
**October 2006**	26	1	4	0	20
**November 2006**	30	1	3	0	17
**December 2006**	27	14	52	32	71
**January 2007**	172	18	10	6	16
**February 2007**	30	17	57	37	75
**March 2007**	30	15	50	31	69
**April 2007**	164	4	2	1	6
