# Supplementary material for: Epidemiology of foot-and-mouth disease in Landhi Dairy Colony, Pakistan, the world largest Buffalo colony
Source: Virol J. 2008 Apr 29;5:53. doi: 10.1186/1743-422X-5-53 (PMC2386124; doi:10.1186/1743-422X-5-53)
Supplement: Additional file 2 — ODP values of all 180 tested serum samples. ODP values of all 180 tested serum samples, for serotype specific and NSP ELISA [file 1743-422X-5-53-S2.pdf]

	Sample nr.	O-Manissa	A-Iraq	Asia 1	C	NSP
October 2006	S 1	18	8	13	16	7
	S 2	8	6	16	21	4
	S 3	17	7	12	17	7
	S 4	15	6	12	17	8
	S 5	17	6	13	16	8
	S 6	17	6	12	17	7
	S 7	17	7	13	18	7
	S 8	18	9	15	19	7
	S 9	16	7	13	17	6
	S 10	8	5	15	19	4
	S 11	8	5	15	20	4
	S 12	16	5	12	15	8
	S 13	15	5	12	15	6
	S 14	7	5	15	20	4
	S 15	7	5	15	19	4
	S 16	17	8	13	17	7
	S 17	8	7	21	34	34
	S 18	8	5	17	20	4
	S 19	16	5	13	17	9
	S 20	7	6	31	40	39
	S 21	8	5	16	20	4
	S 22	7	6	27	44	37
	S 23	16	5	12	16	7
	S 24	9	6	17	23	4
	S 25	9	8	19	23	3
	S 26	15	5	12	16	8
	S 27	8	5	16	22	4
	S 28	17	7	13	18	6
	S 29	9	10	18	25	3
	S 30	17	6	12	17	7
November 2006	S 31	7	5	9	11	6
	S 32	9	6	73	89	21
	S 33	7	5	8	11	2
	S 34	7	5	13	15	12
	S 35	8	5	18	23	11
	S 36	8	12	22	28	14
	S 37	8	7	9	13	2
	S 38	8	7	64	83	19
	S 39	8	5	12	15	10
	S 40	6	5	46	56	5
	S 41	8	6	74	87	18
	S 42	6	5	8	11	2
	S 43	7	6	12	15	13
	S 44	8	6	9	13	2
	S 45	9	9	15	14	15
	S 46	7	9	22	25	14
	S 47	8	9	22	26	12
	S 48	103	108	101	122	112
	S 49	8	5	11	17	1
	S 50	8	6	73	88	20
	S 51	7	5	8	11	2
	S 52	8	9	14	17	13
	S 53	9	16	22	24	16
	S 54	8	7	61	84	20
	S 55	8	7	68	88	22
	S 56	8	7	69	81	17
	S 57	7	6	13	14	13
	S 58	6	5	8	11	2
	S 59	8	5	12	16	2
	S 60	9	7	59	73	22

December 2006	S 61	9	7	4	13	15
	S 62	8	6	10	13	10
	S 63	7	35	24	17	3
	S 64	8	8	9	28	8
	S 65	8	7	4	13	15
	S 66	8	7	34	16	8
	S 67	6	8	4	16	5
	S 68	8	6	9	15	9
	S 69	7	8	4	13	5
	S 70	8	7	32	15	9
	S 71	7	8	5	12	5
	S 72	8	8	9	30	9
	S 73	7	8	5	13	5
	S 74	7	7	10	26	8
	S 75	7	8	4	13	4
	S 76	6	8	5	14	6
	S 77	7	35	25	18	3
	S 78	7	8	10	28	7
	S 79	6	8	5	13	5
	S 80	9	8	32	16	8
	S 81	8	7	9	29	8
	S 82	8	6	32	16	7
	S 83	7	8	4	13	5
	S 84	8	7	34	13	7
	S 85	7	8	5	17	5
	S 86	8	8	32	15	7
	S 87	7	8	5	17	5
	S 88	8	9	5	15	5
	S 89	7	8	4	13	4
	S 90	9	8	10	16	7
January 2007	S 1	6	5	29	13	4
	S 2	6	5	18	17	7
	S 3	6	7	5	29	6
	S 4	10	9	3	25	8
	S 5	7	5	4	39	6
	S 6	5	5	5	15	10
	S 7	5	7	9	18	6
	S 8	6	7	3	14	3
	S 9	8	8	3	10	3
	S 10	6	8	10	34	34
	S 11	8	6	65	51	9
	S 12	6	5	8	12	3
	S 13	5	5	10	11	2
	S 14	5	4	2	8	3
	S 15	8	7	32	20	2
	S 16	6	6	3	22	4
	S 17	10	5	4	14	11
	S 18	7	5	24	28	7
	S 19	6	10	34	24	6
	S 20	6	5	2	29	20
	S 21	5	4	2	10	7
	S 22	7	5	6	40	57
	S 23	5	5	14	9	5
	S 24	6	7	12	26	6
	S 25	8	6	4	24	19
	S 26	5	5	4	15	2
	S 27	6	4	7	28	4
	S 28	12	10	65	45	6
	S 29	8	7	10	16	9
	S 30	7	5	40	39	23

Febuary 2007	S 31	6	6	3	13	3
	S 32	6	5	9	13	8
	S 33	6	4	2	21	2
	S 34	5	5	9	13	8
	S 35	6	5	2	11	17
	S 36	5	5	5	19	65
	S 37	8	7	46	23	30
	S 38	6	5	32	26	3
	S 39	24	5	37	29	12
	S 40	6	6	7	20	9
	S 41	9	5	2	8	11
	S 42	6	5	3	10	2
	S 43	4	4	2	11	11
	S 44	5	5	24	14	3
	S 45	6	5	3	10	2
	S 46	5	5	4	42	3
	S 47	6	5	2	18	3
	S 48	5	5	3	11	2
	S 49	5	5	23	16	2
	S 50	6	5	2	15	7
	S 51	7	5	7	21	20
	S 52	5	6	2	13	2
	S 53	7	6	18	31	2
	S 54	6	5	2	10	7
	S 55	7	8	4	12	16
	S 56	5	5	6	10	6
	S 57	7	6	30	23	9
	S 58	7	5	7	22	21
	S 59	6	5	2	15	7
	S 60	6	5	12	18	2
March 2007	S 61	6	6	9	11	3
	S 62	6	5	4	22	6
	S 63	6	5	12	14	23
	S 64	6	6	20	32	5
	S 65	7	30	7	33	45
	S 66	5	6	2	11	7
	S 67	6	6	27	25	3
	S 68	6	5	26	16	6
	S 69	6	6	4	23	14
	S 70	6	5	2	9	20
	S 71	5	5	18	31	5
	S 72	5	6	3	20	4
	S 73	9	7	3	30	4
	S 74	6	7	6	19	21
	S 75	6	6	27	23	3
	S 76	6	5	5	16	3
	S 77	6	5	3	20	5
	S 78	6	5	12	15	2
	S 79	6	6	15	16	185
	S 80	9	7	2	27	4
	S 81	6	8	3	16	26
	S 82	5	5	6	10	6
	S 83	10	7	4	41	21
	S 84	5	5	6	10	7
	S 85	5	6	16	29	3
	S 86	8	6	3	27	11
	S 87	5	5	16	22	7
	S 88	5	6	7	20	7
	S 89	6	6	5	19	5
	S 90	6	4	7	14	4
